# Obstetric Complications and Polygenic Risk Score: Which Role in Predicting a Severe Short-Term Outcome in Psychosis?

**DOI:** 10.3390/genes12121895

**Published:** 2021-11-26

**Authors:** Sarah Tosato, Chiara Bonetto, Evangelos Vassos, Antonio Lasalvia, Katia De Santi, Margherita Gelmetti, Doriana Cristofalo, Alexander Richards, Mirella Ruggeri, on behalf of the PICOS-Veneto Group

**Affiliations:** 1 Department of Neuroscience, Biomedicine and Movement Sciences, Section of Psychiatry, University of Verona, 37134 Verona, Italy; chiara.bonetto@univr.it (C.B.); antonio.lasalvia@univr.it (A.L.); margherita.gelmetti@aulss9.veneto.it (M.G.); doriana.cristofalo@univr.it (D.C.); mirella.ruggeri@univr.it (M.R.); 2 Social, Genetic and Developmental Psychiatry Centre, Institute of Psychiatry, Psychology & Neuroscience, King’s College London, London SE5 8AF, UK; evangelos.vassos@kcl.ac.uk; 3 The National Institute for Health Research, Maudsley Biomedical Research Centre at South London and Maudsley NHS Foundation Trust and King’s College London, London SE5 8AF, UK; 4 Unit of Psychiatry, Azienda Ospedaliera Universitaria Integrata, 37134 Verona, Italy; katia.desanti@univr.it; 5 MRC Centre for Neuropsychiatric Genetics and Genomics, Division of Psychological Medicine and Clinical Neurosciences, School of Medicine, Cardiff University, Cardiff CF24 4HQ, UK; RichardsAL1@cardiff.ac.uk

**Keywords:** psychosis, polygenic risk score, outcome, obstetric complications

## Abstract

Understanding and improving the outcomes of psychosis remains a major challenge for clinical research. Obstetric complications (OCs) as a risk factor for schizophrenia (SZ) have been investigated as a potential predictor of outcomes in relation to illness severity and poorer treatment outcome, but there are less reports on first episode psychosis (FEP) patients. We test whether OCs, collected in a cohort of FEP patients, can predict illness course and psychopathology severity after 2 years from the onset. Moreover, we explore whether the SZ-polygenic risk score (PRS) would predict the illness course and whether the interaction between OCS and PRS shows a significant effect. A cohort of 264 FEP patients were assessed with standardized instruments. OCs were recorded using the Lewis–Murray scale in interviews with the patients’ mothers: 30% of them reported at least one OC. Patients with at least one OC were more likely to have a non-remitting course of illness compared to those without OCs (35.3% vs. 16.3%, *p* = 0.014). No association between SZ-PRS and course of illness nor evidence for a gene–environment interaction was found. In our sample, poor short-term outcomes were associated with OCs, while SZ-PRS was not a prognostic indicator of poor outcomes.

## 1. Introduction

Understanding and improving the outcomes of psychosis remains a major challenge for clinical research [1]. The search for consistent and reliable prognostic factors that could identify, at the illness onset, which patients will recover completely from those who will not has become an important goal [2]. The final aim of finding prognostic factors is to allow the identification, ideally from illness onset, of those patients with a good outcome from those with a poor one, with the hope that this will have important implications for illness management and tailored treatment.

Obstetric Complications (OCs) are one of the most replicated risk factors for psychosis [3,4]. They have been investigated as a potential predictor of outcomes [5,6] in relation to illness severity and poorer treatment outcomes. Reports of an early age at onset [7,8] and poor premorbid psychosocial adjustment [9] in patients experiencing OCs suggest that neurodevelopmental factors, such as OCs, are etiologically important for at least some patients with schizophrenia [10,11,12]. Interestingly, each of these clinical characteristics is associated with poor prognosis in schizophrenia, implying that OCs themselves are associated with a severe form of illness. Specifically, it has been speculated that OCs might be one source of early cerebral insult, through hypoxic mechanisms [13] leaving behind subtle deficits in widely distributed neuronal circuits, that can place the individual at an increased risk of psychosis during later stages in life [14]. In fact, a reduction in grey and white matter in the brains of individuals who were born preterm, similar to those described in the brains of patients with psychosis, has been found [15,16]. However, despite several indirect sources of evidence of a relationship between a history of OCs and poor prognosis in schizophrenia, to our knowledge, there are no studies testing this hypothesis in first episode psychosis (FEP) patients with prospective follow-up.

Alongside the contribution given by environmental risk factors such as OCs, the genetic contribution on the risk of developing schizophrenia was amply demonstrated in a previous study [17]. Recently, the genetic risk was summarized through the polygenic risk score (PRS), a weighted sum of the number of SNPs, based on the estimated SNP effect sizes obtained from GWAS summary statistics [18]. While many studies have shown that PRS can predict psychosis status [19,20] also in FEP [21], there is no strong evidence supporting a PRS–environment interaction [22]. The schizophrenia-PRS (SZ-PRS) was found to be associated with psychopathological dimensions [23], but not with the duration of psychosis [24] in FEP or with the cognitive decline in the general population [25]. Finally, a potential clinical utility of PRS would be the prediction of severity or response to treatment. PRS was found to be increased in treatment-resistant SZ patients [26] and associated with worse antipsychotic drug efficacy in FEP [27]. Thus, PRS could be useful to identify more severe forms of psychosis, thus having a significant implication for prognosis and treatment [28]. Specifically, considering the heterogeneous outcomes of schizophrenia [29], PRS may help in choosing tailored and appropriate treatment to those who will need it most.

To our knowledge, this is the first longitudinal study aiming at evaluating the relationship between obstetric complications (OCs) and clinical outcomes after 2 years from onset. Specifically, the purpose is to explore whether OCs may be associated with clinical outcomes (illness course and response to treatment) in a sample of FEP patients. In a secondary, exploratory analysis, we investigated whether the SZ-PRS would be associated with illness course and whether there is an interaction between OCS and PRS in predicting illness course.

## 2. Materials and Methods

Based on the WHO 10-Country study [30], this study recruited patients presenting with their first episode of psychosis to Community Mental Health Centres (CMHCs) in the Veneto Region, North-eastern Italy [2].

Inclusion criteria were: (a) age 18–54 years; (b) residence within the catchment areas; (c) presence of at least one of the following symptoms: hallucinations, delusions, qualitative speech disorder, qualitative psychomotor disorder, and bizarre or grossly inappropriate behavior; or two of the following symptoms: loss of interest, initiative, and drive; social withdrawal; episodic severe excitement; purposeless destructiveness; overwhelming fear; or marked self-neglect; and (d) first lifetime contact with CMHCs, prompted by these symptoms. Exclusion criteria were: (a) prescribed antipsychotic medication (>3 months); (b) mental disorder due to general medical condition; (c) moderate–severe intellectual disability assessed by clinical functional assessment.

Written informed consent, including permission to contact their relatives, was obtained after a complete description of the study, which was approved by both the Ethics Committee of the coordinating centre and the local Ethics Committees of participating sites.

Patients were also assessed after 2 years: those still in contact with services were approached through their treating clinicians, while those no longer in contact were contacted by their former treating clinicians. Patients who had left the area of residence were traced by contacting family members or their general practitioner. The follow-up assessments included face-to-face interviews with participants when possible, and with family members and the treating psychiatric teams, as well as the perusal of clinical notes.

Details on the study design, sample recruitment and representativeness, and clinical assessment were previously published [31].

### 2.1. Measures

At both baseline and follow-up, patients were assessed with a set of standardized measures including the Positive and Negative Symptoms Scale (PANSS) [32] and the Global Assessment of Functioning (GAF) [33]. Diagnosis was confirmed after six months from inclusion using the Item Group Checklist (IGC) of the SCAN [34], which allows for the rating of information derived from case records integrated with interviews with the patient case manager if needed. We included only ICD-10 diagnostic codes of psychosis (F1x.04; F1x.5; F1x.7; F20–29; F30–31.9; F32–33), categorised as follows: SZ (F20; F21, F25), “other non-affective psychosis” (F1x.4, F1x.5; F1x.7; F22, F23, F29), and “affective psychosis” (F30–31.9; F32–33).

Information about Obstetric Complications (OCs) was collected at baseline by interviewing the mother of patients using the Lewis–Murray scale [35]. The scale rates 15 obstetric complications as absent or definitely present; 9 of the exposures can also be rated as equivocally present. We considered either definite or equivocal exposure to any complication of pregnancy or labor as positive exposure [36]. Patients were divided according to the number of OCs in three groups: 0 OCs, 1–2 OCs, 3–5 OCs.

Information on illness course was obtained at 2-year follow up with the WHO Life Chart [37], based on case notes and clinical interviews whenever possible. It defines the illness course type as either: continuous (no remission of symptoms of greater than 6 months); episodic (one or more period of remission of at least 6 months, and no episode of psychosis, including the first one, that lasted for 6 months or more); or intermediate (never achieved sustained periods of remission, but also never experienced psychotic symptoms for prolonged periods). We classified patients by defining Non-Remitters as those with a continuous or intermediate illness course and Remitters as those with an episodic illness course.

Moreover, we defined responders to treatment [38] as patients with a >50% reduction in their baseline PANSS score, using the following formula applied to all PANSS dimensions: ((PANSS at baseline − PANSS at follow up)/PANSS at baseline) × 100. In addition to the previous measures, we defined patients as functionally improved at 2-year follow-up if there was 50% GAF improvement using the following formula applied to all PANSS dimensions: ((GAF at follow up − GAF at baseline)/GAF at baseline) × 100.

### 2.2. Polygenic Risk Score Calculation

At baseline, venous blood samples (15 mL) were collected in Ethylenediaminetetraacetic acid (EDTA) containing tubes from each participant, and DNA was extracted from blood leukocytes by using a commercial kit (ABgene, Blenheim Road, Epson, Surrey, UK). Patients were genotyped at the Institute of Psychological Medicine and Clinical Neurology, Cardiff University, Cardiff, UK, using custom Illumina HumanCoreExome-24 BeadChip genotyping arrays containing probes for 570,038 genetic variants (Illumina, San Diego, CA, USA). Genotype data were called using the GenomeStudio package 2.0 and transferred into PLINK format for further analysis using PLINK 1.9.

Quality control was conducted in PLINK v1.07 [39] or with custom Perl scripts. Variants with call rates <98% or with Hardy–Weinberg Equilibrium *p*-values < 10^−6^ were excluded from the dataset. After QC, 559,505 variants remained.

Samples with call rates <98% were excluded from the dataset. A linkage disequilibrium pruned set of variants was calculated using the -indep-pairwise command in PLINK (maximum r2 = 0.25, window size = 500 SNPs, window step size = 50 SNPs) and used for further analyses. Homozygosity F values were calculated using the-het command in PLINK, and outlier samples (F < −0.11 or F > 0.15) were excluded. The genotypic sex of samples was calculated from X chromosome data using the -check-sex command in PLINK, and samples with different genotypic sex to their database sex were excluded.

Using the clumping and thresholding method, PRS was built at 10 *p*-value thresholds, using PRSice software v2.3 [40], based on the summary data from the last Psychiatric Genomics Consortium (PGC) schizophrenia GWAS including SNPs with minor allele frequency (MAF) >0.1 [41]. A principal component analysis (PCA) was implemented in PRSice and the first ten PCs were included as covariates in the genetic analyses to control for the effects of population stratification. For our analyses, we preselected PRS at the 0.1 *p*-value threshold.

### 2.3. Statistical Analyses

Descriptive data were presented as means and standard deviations for continuous variables or frequencies and percentages for categorical variables. Categorical variables were compared using the Chi-square test. Continuous variables were compared by t test for independent groups, or by ANOVA. A logistic regression model for the follow-up illness course was estimated in order to explore the possible interaction between OCs and PRS.

All p-values were two-tailed with an accepted significance level of 0.05, with no multiple testing correction applied due to the explorative nature of the study. Analyses were performed using SPSS 22.0 or Stata 15.

## 3. Results

**Figure 1:**
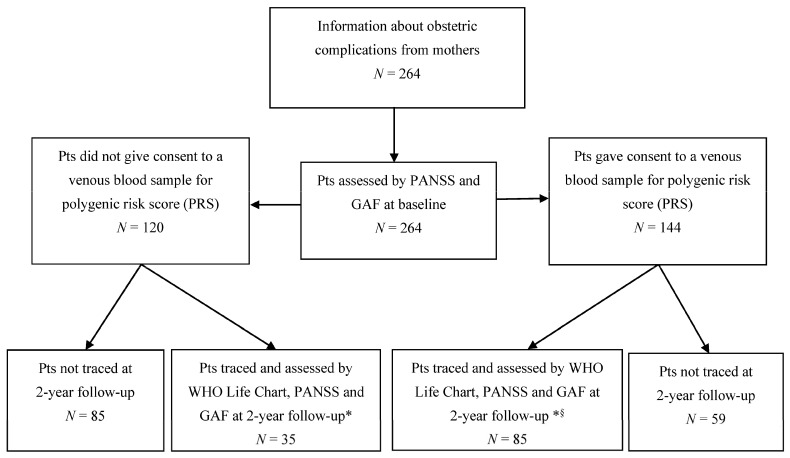
Flow-chart of assessments for the sample (*N* = 264). * *n* = 120 patients with PANSS and GAF at both baseline and 2-year follow-up and with WHO Life Chart at 2-year follow-up (sample for clinical assessments). § *n* = 85 patients with clinical assessments and polygenic risk score (sample for GxE).

At baseline, 264 (91.7%) mothers accepted to be interviewed for OCs and constituted the study cohort. In Figure 1, we report a flow diagram of patients’ assessments. 

Out of the 264 patients (with OCs data available), 186 (70%) patients did not experience OCs, whereas 78 (30%) patients reported at least one definite OC. Out of 78 patients with at least one OC, 74.3% (N = 58) patients experienced 1–2 OCs and 25.7% (*N* = 20) experienced 3–5 OCs.

Those patients with the highest number of OCs (3–5) showed a significantly higher prevalence of males (10.8%), an earlier age of onset (27.8 sd 8.7), a higher prevalence of schizophrenia (16.4%), and more severe levels of negative symptoms (3.69 sd 1.52) (Table 1).

### 3.1. Obstetric Complications and Outcomes

At 2 years, 120 patients were traced (see Supplementary Files for details). Of these, 26 (21.7%) fulfilled criteria for a non-remitting course of illness, and 94 (78.3%) for a remitting course. No significant differences in socio-demographic and clinical characteristics were found between those followed for 2 years and those lost to follow-up. We found that patients with at least 1 OC were more likely to have a non-remitting course of illness, compared to patients without OCs (35.3% vs. 16.3%, *p* = 0.014) (Table 2).

Regarding response to treatment, we found that the group of patients with no OCs had a higher improvement in the negative PANSS dimension with respect to patients with 1–2 or 3–5 OCs (61.4% vs. 15.9% vs. 22.9%, *p* = 0.005). There were no other significant differences in other PANSS dimensions or functioning (Table 3).

### 3.2. OCs and Genetics

In the subgroup with PRS and course of illness data at 2 years (*N* = 88) (see Supplementary Files for details), we found no association between PRS and course of illness.

The gene–environment interaction was explored using a linear logistic regression model, with the course of illness at the 2-year follow-up as the dependent variable and the two main effects together with obstetric complications by PRS interaction as independent variables. No evidence of gene–environment interaction was found (Table 4).

## 4. Discussion

This is the first longitudinal study aiming at evaluating the relationship between obstetric complications (OCs) and clinical outcomes over a 2-year period. We found that patients with at least one OC were more likely to have a non-remitting course of illness after 2-years. Because the early phase of psychosis represents a critical period during which experiencing or not experiencing a relapse could have a strong impact on recovery [31], our finding that OCs are associated with a non-remitting course after 2 years suggest that OCs could identify, at the onset, those patients with a worse outcome. Since the onset of psychosis normally occurs during the young adult period, which is characterised by the most pronounced individual social, cultural, and economic development, it is important to identify, with objective and easy-to-collect measures such as OCs, those patients who will need more extensive therapeutic support in order to improve their outcomes.

In our sample, we found that patients with the highest number of OCs (3–5) had a significantly higher prevalence of males, had more frequent diagnoses of schizophrenia, and had earlier ages of onset. These findings are consistent with other studies, which report that OCs are more prevalent in men [42,43,44,45] and in those diagnosed with schizophrenia [46,47], and are associated with earlier age at onset [48,49,50,51]. It was suggested that gender difference in obstetric history could be a relevant factor in accounting for the earlier onset and poorer outcomes of schizophrenia in male patients [52]. A confounding factor in the association between OCs and early age at onset could be gender itself, since most studies have found that age at onset of psychosis differs according to gender, where women tend to be a few years older than men at first admission [52]. The age distribution in females, with a second peak in incidence at menopausal age, suggested that women’s distribution of schizophrenia onset could be an oestrogen effect. Indeed, the secretion of this hormone declines rapidly when women reach menopause. On the other hand, testosterone increases at a young age, but it shows no rapid decline when compared to oestrogen during menopausal age. An explanation might also be that testosterone could be capable of speeding up the manifestation of psychosis, working in the opposite direction of oestrogen. These hypotheses were previously tested using animal models and it was revealed that oestrogen accounts for the gender difference in age of schizophrenia onset [53,54].

We also found that patients with the highest number of OCs presented more severe levels of negative symptoms at baseline. Interestingly, at follow-up, the only PANSS dimension discriminating patients with improvement in psychopathology from those without was the negative dimension. Specifically, we found that the group of patients with no OCs had higher improvement in the negative PANSS dimension compared to patients with OCs. The length of remission of negative symptoms was demonstrated to be by far the strongest predictor of functional outcomes at 1- and 2-year follow-up in patients experiencing the early phase of psychosis [55]. Negative symptoms contribute to the burden of psychosis, not only for the patients but also for families and society [56], and they are the symptoms that treatments have shown a limited impact on. The fact that OCs are associated with more severe negative symptoms both at onset and during the early stage of psychosis could help clinicians to identify those patients who require intensive treatment.

We also found no association between PRS and course of illness. There is growing interest in the predictive role of PRS on symptom trajectories. As for common disorders, such as diabetes type 2, coronary heart disease, and breast cancer, in which PRS is already revealing potential clinical utility, it was suggested that PRS for psychoses could perform the same function [18]. A previous work found that schizophrenia PRS predisposes individuals to worse course-of-illness severity, avolition, and cognitive deficits [28]. In our sample, PRS for schizophrenia seems not to represent a prognostic marker of poor outcome, probably indicating that our study was underpowered for this association due to the small sample size. Furthermore, no evidence of gene–environment interaction was found in either group, remitting and non-remitting course. This finding is line with a recent large-scale study, which included our sample, that did not find evidence of a role of SZ-PRS caused by the interaction of OCs in the prediction of schizophrenia case-control [57]. Our report, although of an exploratory nature, is part of the research aiming to examine gene–environment interactions in general and in relation to a specific phenotype—remission. Our patients had extensive clinical and biological assessments at baseline, were treated according to a specific medication algorithm, and were followed up prospectively with standardized assessments. This design allowed us to closely monitor symptom recurrences and to accurately define clinical outcomes (i.e., the course of illness). Moreover, we accurately defined the exposition to the environmental risk factors through the interviews with the patients’ mothers. Despite these methodological devices, studies addressing GxE interactions comprise distinct models underlying various scenarios of causality. For example, the genotype could increase the effect of environmental exposure, remaining silent when environmental exposure does not act. Conversely, the genotype may affect the phenotype in the presence of an environmental risk factor, but when the risk genotype is not present, a higher level of that environmental factor is required to increase risk. In another GxE model, the environmental factor increases the effects only of the high-risk genotype, but not of the low-risk genotype [58]. The effects of GxE interactions are even more insidious in psychiatric research; indeed, it could be suggested that some risk genotypes could make individuals more likely to be involved in high-risk environments [59]. Another limitation of these studies relates to their methodological heterogeneity, as well as a variety of explored outcome variables. Among these, one of the main methodological problems of studies addressing GxE is sample size [59]. It was demonstrated, for example, that an interaction of moderate effect size with the genotype that is present in only 5% of people would need a sample of 5200 individuals to reach the power of 80% [60]. These could explain the different and contrasting results.

Our results should be interpreted taking into account several strengths. First, our study was conducted by examining a large epidemiologically based cohort of FEP patients, composed of both affective and non-affective psychosis, reducing the probability of selection bias due to diagnostic sampling. Second, there was no attempt by the researcher to influence psychotropic treatment, providing an accurate picture of the routine treatment of out-patients. Third, this study monitored the course of illness over the short term in an area with relatively low mobility. Fourth, it was carried out via “real world” public mental health services that have been operational for several years, an approach that obviated the limitations of research programmes conducted in dedicated research centres. Finally, in our sample, OCs were collected by interviewing the mother of the patients, so the likelihood to underestimate the recall of OCs is small. Specifically, there is evidence that maternal reports about OCs are consistent with those collected in medical records [61].

This study also has some limitations. Our study substantially recruited FEP patients who had been treated within the public sector; it is, therefore, likely that the patients who approached the private sector may have been excluded. However, this should not be considered a major problem, since it was previously shown that, in the Veneto Region, only a negligible fraction of patients affected by psychosis were treated in the private sector and that it is standard practice for a GP to refer all psychosis cases to the public mental health services [62]. In addition, as discussed previously, our sample is small for genetic analysis, so the negative results regarding the PRS prediction of illness course could reflect the low power of our sample. Finally, it needs to be clarified that SZ-PRS explains about 7% of the variance in the liability scale. Future, more powerful, PRSs will have higher predictive ability for these types of analyses.

## 5. Conclusions

Our study suggests that the presence of OCs is associated with poor illness course and less improvement of negative symptoms at 2-year follow-up. We did not find an association between PRS and course of illness or evidence of gene–environment interaction.

Relapse prevention is a major challenge in the care of patients with schizophrenia. It is well established that each relapse is a traumatic experience, associated with potentially serious psychosocial and functional consequences that impact the quality of life of patients, families, and caregivers. Our opportunity to study the course of illness and its predictors arose in the context of a long-term study of first episodes of psychosis.

Identifying patients’ outcome trajectories and the potential risk factors linked with a worse course of illness is a priority, because this kind of information could lead to tailored treatment and effective preventive interventions resulting in less risk of relapse. Finally, building a method to predict relapse with adequate accuracy could help in terms of implementing interventions to minimize relapses and their consequences.

## Figures and Tables

**Table 1:** Demographic and clinical differences across patients with increasing numbers of OCs (*N* = 264). X^2^(df), *p* stays for the Chi-square test (degree of freedom) with its *p*-value; F(df1,df2), *p* stays for the F test (numerator degree of freedom, denominator degree of freedom) with its *p*-value.

	No OCs	1–2 OCs	3–5 OCs	X^2^ or ANOVA
**Gender, *N* (%)**				Χ^2^(2) = 9.062, *p* = 0.011
Male	94 (63.5%)	38 (25.7%)	16 (10.8%)	
Female	92 (79.3%)	20 (17.2%)	4 (3.4%)	
**Age at onset, years (sd)**	31.9 (9.5)	29.2 (9.1)	27.8 (8.7)	F(2, 261) = 3.133, *p* = 0.045
**Diagnosis, *N* (%)**				Χ^2^(4) = 11.479, *p* = 0.022
Schizophrenia (SCZ)	40 (65.6%)	11 (18.0%)	10 (16.4%)	
Affective psychosis	44 (80.0%)	10 (18.2%)	1 (1.8%)	
Other non-affective psychosis	102 (68.9%)	37 (25.0%)	9 (6.1%)	
**Family history, *N* (%)**				Χ^2^(4) = 2.641, *p* = 0.620
Positive	61 (80.0%)	23 (20.0%)	11 (11.6%)	
Negative	97 (70.8%)	31 (22.6%)	9 (6.6%)	
Unknown	4 (80.0%)	1 (20.0%)	0 (0%)	
**PANSS, mean (sd)**				
Positive	3.10 (1.03)	3.01 (1.05)	2.89 (.98)	F(2, 261) = 0.494, *p* = 0.611
Negative	2.56 (1.38)	2.24 (1.05)	3.69 (1.52)	F(2, 260) = 8.804, *p* < 0.001
General	2.64 (.75)	2.52 (.60)	2.82 (.84)	F(2, 260) = 1.315, *p* = 0.270
Total	2.73 (.76)	2.57 (.57)	3.04 (.84)	F(2, 261) = 3.039, *p* = 0.050
**GAF, *N* (%)**	38.78 (10.98)	38.60 (10.40)	37.75 (9.62)	F(2, 260) = 0.084, *p* = 0.920

**Table 2:** Association between illness course at 2 years and OCs (N = 120).

	2-Year Follow-up		
	Remitter *N* = 94	Non-Remitter *N* = 26	Fisher’s Exact Test
**No OC** **s**	72 (83.7%)	13 (16.3%)	0.014
**At least 1 OCs**	22 (64.7%)	13 (35.3%)	

**Table 3:** Association between OCs and response to treatment and functioning after 2 years (*n* = 120).

Improvement after 2 yrs	No OCs	1–2 OCs	3–5 OCs	X^2^
PANSS, *N* (%) *				
Positive < 50	25 (61%)	12 (29.3%)	4 (9.8%)	Χ^2^(2) = 1.221, *p* = 0.543
Positive ≥ 50	60 (68.2%)	18 (20.5%)	10 (11.4%)	
Negative < 50	58 (68.2%)	23 (27.1%)	4 (4.7%)	Χ^2^(2) = 10.434, *p* = 0.005
Negative ≥ 50	27 (61.4%)	7 (15.9%)	10 (22.7%)	
General < 50	51 (61.4%)	22 (26.5%)	10 (12.0%)	Χ^2^(2) = 2.062, *p* = 0.357
General ≥ 50	34 (73.9%)	8 (17.4%)	4 (8.7%)	
Total < 50	51 (63.7%)	22 (27.5%)	7 (8.8%)	Χ^2^(2) = 2.475, *p* = 0.290
Total ≥ 50	32 (68.1%)	8 (17.0%)	7 (14.9%)	
**GAF, N (%) §**				
<50	32 (69.6%)	10 (21.7%)	4 (8.7%)	Χ^2^(2) = 0.658, *p* = 0.719
≥50	51 (63.0%)	20 (24.7%)	10 (12.3%)	

* Patients were clinically improved at 2-year follow-up regardless of whether there was a 50% PANSS reduction. § patients were functionally improved at 2-year follow-up regardless of whether there was 50% GAF improvement.

**Table 4:** Logistic regression results for GxE interaction (*n* = 88).

	Remitter FU2	
	OR	*p*-Value
At least 1 OC	2.20	0.213
SCZ-PRS	1.28	0.560
Interaction	2.39	0.205

## Data Availability

The data supporting the findings are not publicly available, but they can be provided by the corresponding author (ST) on reasonable request.

## Supplementary Materials

Comparisons between the 120 patients who did not give consent to a venous blood sample and the 144 patients who consented to give a venous blood sample are available online at https://www.mdpi.com/article/10.3390/genes12121895/s1, Table S1: Differences in clinical assessments (PANSS and GAF) at baseline in the two groups (120 vs. 144), Table S2: Differences in having had at least one obstetric complication in the two groups (120 vs. 144), Table S3: Differences in clinical assessments (PANSS and GAF) at baseline in the two groups (85 vs. 35), Table S4: Differences in having had at least one obstetric complication in the two groups (85 vs. 35), Table S5: Differences in clinical assessments (PANSS and GAF) at baseline in the two groups (59 vs. 85), Table S6: Differences in having had at least one obstetric complication in the two groups (59 vs. 85).
