# Supplementary material for: Obstetric Complications and Polygenic Risk Score: Which Role in Predicting a Severe Short-Term Outcome in Psychosis?
Source: Genes (Basel). 2021 Nov 26;12(12):1895. doi: 10.3390/genes12121895 (PMC8702213; doi:10.3390/genes12121895)
Supplement: Supplementary file 1 [file genes-12-01895-s001.zip › genes-1461582-supplementary.pdf]

Differences between completers and non-completers on assessments

Comparisons between the 120 patients who did not give consent to a venous blood sample and the 144 patients who consented to give a venous blood sample

Table S1. Differences in clinical assessments (PANSS and GAF) at baseline in the two groups (120 vs 144)

Clinical assessment at baseline	Consent to a venous blood sample	Mean (sd)	p-value t test for 2 independent samples
PANSS positive	No	3.24 (1.07)	0.013
	Yes	2.92 (0.98)	
PANSS negative	No	2.54 (1.35)	0.610
	Yes	2.63 (1.38)	
PANSS general	No	2.59 (0.63)	0.433
	Yes	2.66 (0.81)	
PANSS total	No	2.73 (0.67)	0.903
	Yes	2.72 (0.79)	
GAF	No	38.8 (10.7)	0.979
	Yes	38.8 (10.5)	

Table S2. Differences in having had at least one obstetric complication in the two groups (120 vs 144)

	Consent to a venous blood sample		p-value Fisher's exact test
**Obstetric complications**	No	Yes	
No	84 (70.0%)	97 (67.4%)	0.691
At least 1	36 (30.0%)	47 (32.6%)	

Comparisons between the 85 patients who were not traced and assessed at the 2-year follow-up and the 35 patients who were assessed (within the 120 patients who did not give the consent to a venous blood sample)

Table S3. Differences in clinical assessments (PANSS and GAF) at baseline in the two groups (85 vs 35)

**Clinical assessment at baseline**	**Assessed and 2-yr follow-up**	**Mean (sd)**	**p-value t test for 2 independent samples**
PANSS positive	No	3.23 (1.07)	0.889
	Yes	3.26 (1.09)	
PANSS negative	No	2.61 (1.41)	0.378
	Yes	2.37 (1.20)	
PANSS general	No	2.56 (0.58)	0.510
	Yes	2.65 (0.74)	
PANSS total	No	2.73 (0.65)	0.964
	Yes	2.72 (0.73)	
GAF	No	39.5 (10.4)	0.232
	Yes	36.9 (11.4)	

Table S4. Differences in having had at least one obstetric complication in the two groups (85 vs 35)

	**Assessed at 2-year follow-up**		**p-value Fisher's exact test**
**Obstetric complications**	No	Yes	
No	56 (65.9%)	28 (80.0%)	0.188
At least 1	29 (34.1%)	7 (20.0%)	

Comparisons between the 59 patients who were not traced and assessed at the 2-year follow-up and the 85 patients who were assessed (within the 144 patients who consented to give a venous blood sample)

Table S5. Differences in clinical assessments (PANSS and GAF) at baseline in the two groups (59 vs 85)

Clinical assessment at baseline	Assessed and 2-yr follow-up	Mean (sd)	p-value t test for 2 independent samples
PANSS positive	No	2.76 (0.91)	0.094
	Yes	3.03 (1.01)	
PANSS negative	No	2.65 (1.40)	0.849
	Yes	2.61 (1.38)	
PANSS general	No	2.66 (0.82)	0.979
	Yes	2.66 (0.80)	
PANSS total	No	2.69 (0.75)	0.722
	Yes	2.74 (0.82)	
GAF	No	39.8 (11.3)	0.321
	Yes	38.1 (10.0)	

Table S6. Differences in having had at least one obstetric complication in the two groups (59 vs 85)

	Assessed at 2-year follow-up		p-value Fisher's exact test
**Obstetric complications**	No	Yes	
No	39 (66.1%)	58 (68.2%)	0.857
At least 1	20 (33.9%)	27 (31.8%)	

Conclusions

The analyses comparing the different groups (consent to a venous blood sample and clinical assessment at the 2-year follow-up, respectively) showed that the only statistical significant difference was in PANSS positive symptoms for patients who consented and did not consent to give

a venous blood sample (p=0.013, Table 1). In detail, patients who did not consent had higher levels of positive symptoms compared to those who consented (3.24 sd 1.07 vs 2.92 sd 0.98).
